# Osteoclastic differentiation and resorption is modulated by bioactive metal ions Co^2+^, Cu^2+^ and Cr^3+^ incorporated into calcium phosphate bone cements

**DOI:** 10.1371/journal.pone.0182109

**Published:** 2017-08-01

**Authors:** Anne Bernhardt, Martha Schamel, Uwe Gbureck, Michael Gelinsky

**Affiliations:** 1 Centre for Translational Bone, Joint and Soft Tissue Research, University Hospital *Carl Gustav Carus* and Faculty of Medicine of Technische Universität Dresden, Dresden, Germany; 2 Department for Functional Materials in Medicine and Dentistry, University of Würzburg, Würzburg, Germany; Universite de Nantes, FRANCE

## Abstract

Biologically active metal ions in low doses have the potential to accelerate bone defect healing. For successful remodelling the interaction of bone graft materials with both bone-forming osteoblasts and bone resorbing osteoclasts is crucial. In the present study brushite forming calcium phosphate cements (CPC) were doped with Co^2+^, Cu^2+^ and Cr^3+^ and the influence of these materials on osteoclast differentiation and activity was examined. Human osteoclasts were differentiated from human peripheral blood mononuclear cells (PBMC) both on the surface and in indirect contact to the materials on dentin discs. Release of calcium, phosphate and bioactive metal ions was determined using ICP-MS both in the presence and absence of the cells. While Co^2+^ and Cu^2+^ showed a burst release, Cr^3+^ was released steadily at very low concentrations (below 1 μM) and both calcium and phosphate release of the cements was considerably changed in the Cr^3+^ modified samples. Direct cultivation of PBMC/osteoclasts on Co^2+^ cements showed lower attached cell number compared to the reference but high activity of osteoclast specific enzymes tartrate resistant acid phosphatase (TRAP), carbonic anhydrase II (CAII) and cathepsin K (CTSK) and significantly increased gene expression of vitronectin receptor. Indirect cultivation with diluted Co^2+^ cement extracts revealed highest resorbed area compared to all other modifications and the reference. Cu^2+^ cements had cytotoxic effect on PBMC/osteoclasts during direct cultivation, while indirect cultivation with diluted extracts from Cu^2+^ cements did not provoke cytotoxic effects but a strictly inhibited resorption. Cr^3+^ doped cements did not show cytotoxic effects at all. Gene expression and enzyme activity of CTSK was significantly increased in direct culture. Indirect cultivation with Cr^3+^ doped cements revealed significantly higher resorbed area compared to the reference. In conclusion Cr^3+^ doped calcium phosphate cements are an innovative cement modification because of their high cytocompatibility and support of active resorption by osteoclasts.

## Introduction

The development of synthetic calcium phosphate based bone graft materials includes the incorporation of bioactive molecules, like growth factors and drugs to generate osteoinductive properties. Besides organic components, metal ions in low doses are increasingly applied for the modification of bone graft materials to improve osteogenesis and neovascularization [1–3]. Co^**2+**^ doping of calcium phosphate was shown to have proangiogenic effects [4,5]. Cu^**2+**^ modification of a calcium phosphate ceramic resulted in an enhanced and directed vascularization [6]. In a previous study we have analyzed the impact of Co^**2+**^, Cu^**2+**^ and Cr^**3+**^ doping of biomimetic calcium phosphate bone cement on materials properties, as well as on proliferation and osteogenic differentiation of human mesenchymal stromal cells *in vitro* [7]. A positive effect of doping with Cr^**3+**^ and, to a less extend, of Cu^**2+**^ on proliferation and osteogenic differentiation was found.

However, examinations for *in vitro* osteogenic capacity of potential bone graft materials are not sufficient to estimate clinical performance. Since the balance between new bone formation and resorption of the materials is crucial for successful remodeling, the *in vitro* analysis of osteoclast-mediated degradation of the materials is a logical next step to evaluate remodeling of the materials *in vivo*. Brushite forming calcium phosphate bone cements show a higher chemical dissolution rate compared to hydroxyapatite (HA) forming calcium phosphate bone cements [8]. Nevertheless, active resorption of these materials is important to enable remodeling, especially due to the phase conversion of brushite to HA at neutral pH [9].

The effect of Co^**2+**^ and Cr^**3+**^ ions on osteoclast formation and resorption activity is in the focus of interest, since both ions are applied in prosthetic implant alloys. Co^**2+**^ and Cr^**3+**^ are released from wear particles after metal-on-metal joint arthroplasty and the concentration of these ions is elevated in serum and urine of the respective patients [10,11]. It was shown, that metallic wear particles from cobalt-chromium alloys induce the formation of multinucleated giant cells [12]. Co^**2+**^ and Cr^**3+**^ ions released from wear debris are suspected to promote aseptic loosening and osteolysis [13]. Andrews and co-workers examined the influence of Co^**2+**^ and Cr^**3+**^ ions on osteoclast formation and resorption activity and found both stimulatory and inhibitory effect of these ions, depending on the concentration [14]. The effect of Cu^**2+**^ ions incorporated in calcium phosphate films was studied with primary rat osteoclasts and a significantly reduced resorptive activity was reported [15].

Cell-based *in vitro* resorption assays are an important tool to simulate the *in vivo* biodegradation of resorbable bone graft materials to estimate their clinical performance [16]. In a recently published paper we analyzed the significance of osteoclast specific enzyme activities tartrate resistant acid phosphatase (TRAP), carbonic anhydrase II (CAII) and cathepsin K (CTSK) for the evaluation of resorption [17]. The central finding was that the correlation between osteoclast-related enzyme activities and resorption is dependent on the resorbed material. It was postulated, that resorption of inorganic calcium phosphate materials is reflected by the intracellular TRAP and CAII activity, while in the case of collagen containing materials like dentin and mineralized collagen the extracellular TRAP and CTSK activity correlates to the resorbed area [17]. Brushite forming calcium phosphate bone cements display a rough surface [7] and therefore the discrimination between resorption pits and structural irregularities is not possible both on the non-modified brushite cement and the metal ion doped modifications. The experimental setup of the present study involves therefore 1) the direct cultivation of osteoclasts, derived from human peripheral blood mononuclear cells (PBMC) on the different cement modifications and the analysis of the osteoclast specific enzyme activities of TRAP and CAII and the mRNA expression of osteoclast markers and 2) the cultivation of osteoclasts on TCPS and dentin slices in the presence of cement extracts to evaluate the influence of the cement modifications on the resorption activity by classical pit assays and TRAP staining. Furthermore, the passive and active, osteoclast-mediated release of Ca^2+^, PO_4_^3-^ and Cr^3+^ from the cement was examined to analyse the resorption of the Cr^3+^ modified cements.

The aim of the study was to analyse the influence of doping with bioactive metal ions on osteoclastic resorption of calcium phosphate bone cement.

## Materials and methods

### Cement preparation

Brushite-forming calcium phosphate cements modified with low doses of Cu^2+^, Co^2+^ and Cr^3+^ were prepared as described previously [7]. Briefly, dicalcium phosphate anhydrous (DCPA, Baker, Germany) and calcium carbonate (CC, Merck, Germany) were mixed with metal nitrates (Cu(NO_3_)_2_·3 H_2_O; Co(NO_3_)_2_·6 H_2_O; Cr(NO_3_)_3_·9 H_2_O, Aldrich) in an appropriate stoichiometric ratio. The powder mixtures were sintered at 1050°C for 5 h resulting in metal ion doped ß-TCP with a metal ion concentration of 10 and 100 mmol per mol β-TCP. The sintered cakes were crushed and sieved with 100 μm pore size-mesh followed by ball milling for 60 min at 200 rpm. Brushite cements were produced by mixing the β-TCP powder in a molar ratio of 1:0.8 with anhydrous monocalcium phosphate (Ca(H_2_PO4)_2_, MCPA, Aldrich) for 30 s. For the modification with 50 mmol Cr^3+^ per g β-TCP samples of β-TCP powder doped with 100 mmol Cr^3+^ were mixed in an equimolar ratio with pure β-TCP powder. An aqueous solution of 0.1 M phytic acid (Aldrich) was used as setting retarder as liquid cement phase in a powder to liquid ratio of 2 g/ml. The cement pastes were then transferred into silicon moulds with 6 mm diameter and stored in a water bath with 100% relative humidity and 37°C for 24 h. The following cement modifications were included into this study: 10 mmol Cu^2+^/g β-TCP (“Cu10”), 10 mmol Co^2+^/g β-TCP (“Co10”), 10 mmol Cr^3+^/g β-TCP (“Cr10”), 50 mmol Cr^3+^/g β-TCP (“Cr50”) Furthermore, cement without metal ions was prepared as a reference. Prior to use in cell culture experiments, the cement samples were sterilized by γ-irradiation.

### Quantification of ions released by the cements

Ion concentrations (Ca^2+^, P as PO_4_^3-^, Cr^3+^) in the cell culture supernatants and in cement extracts were determined using inductively-coupled-plasma mass-spectrometry (ICP-MS, Varian, Germany). The quantitative measurement was carried out against standard solutions (Merck) containing defined concentrations of all ions of interest.

### Preparation of cement extracts

Cement discs were incubated in α-MEM containing 5% heat-inactivated FCS, 5% human A/B serum (Sigma), 100 U/ml penicillin, 100 μg/ml streptomycin, 2 mM glutamine for 3 weeks. Medium was changed twice weekly and the collected extracts were combined for each cement modification. Concentration of Cu^2+^, Co^2+^, and Cr^3+^ in the combined fraction was analysed by ICP (see above) and were as follows: Cu10: 11286 +/- 311 μg/l (177.6 μM); Co10: 11809 +/- 411 μg/l (200.4 μM), Cr10: 77 +/- 4 μg/l (1.48 μM), Cr50: 160 +/- 5 μg/l (3.08 μM). Before applying the cement extracts to cell culture the extracts from the Cu10 and Co10 cements were diluted 1: 10 with medium, resulting in Co^**2+**^ concentration of 20.0 μM and a Cu^**2+**^ concentration of 17.8 μM.

### Osteoclast formation

Peripheral blood mononuclear cells (PBMC) were differentiated into osteoclasts under stimulation with macrophage colony stimulating factor (MCSF) and Receptor Activator of NF-κB Ligand (RANKL). PBMC were isolated from human buffy coats, purchased from the German Red Cross Dresden, as described previously [18]. Briefly, PBMC were separated by density gradient centrifugation (Ficoll Paque Plus), and remaining erythrocytes were lysed with deionized water for 1 min. Composition of PBMC fraction (containing T-lymphocytes, B- cells and monocytes) was evaluated with an automated cell counter (Scepter, Millipore). Cement discs (6 mm diameter, 1 mm height, sterilized by γ-irradiation and pre-incubated in 1 ml of α-MEM containing 10% heat-inactivated FCS overnight) were seeded with PBMC containing 2*10^5^ monocytes per sample. For indirect cultivation with cements extracts, PBMC containing 4*10^5^ monocytes were seeded on dentin discs of 0.8 mm thickness, which have been cut from canine teeth of minipigs [17] in 48-well tissue culture polystyrene (TCPS) plates. Furthermore, 48-well TCPS plates were seeded with PBMC containing 1*10^5^ monocytes. Direct cultivation of PBMC/osteoclasts on the cements was repeated in four independent experiments: three experiments were performed with PBMC of two donors each, and one experiment with PBMC of three donors. Since only the reference and Cr10 cement were included in all four experiments, the total number of biochemical data included cells of 9 donors (45 individual samples) each for reference and Cr10, cells of 6 donors (30 individual samples) each for Cu10 and Co10 modification and cells of 5 donors (25 individual samples) for Cr50 modification, since all biochemical examinations were done in pentaplicates. Additional samples were prepared for microscopic and gene expression analysis. For indirect cultivation with cement extracts cells of three donors were used in pentaplicates on TCPS (15 individual samples per modification) and in duplicates on dentin (6 individual samples per modification).

Seeding was performed in α-MEM containing 10% heat-inactivated FCS, 100 U/ml penicillin, 100 μg/ml streptomycin und 2 mM glutamine. One day after initial adherence of the monocytes, medium was changed into α-MEM containing 5% heat-inactivated FCS, 5% human A/B serum (Sigma), 100 U/ml penicillin, 100 μg/ml streptomycin, 2 mM glutamine and 25 ng/ml M-CSF (Peprotech). After two days of cultivation, cells were additionally supplemented with 50 ng/mL RANKL (Peprotech). In the case of indirect cultivation extracts of the different cement modifications were added. Medium was changed twice weekly. After 16 days of cultivation, cell seeded cement and dentine discs were washed twice with PBS and frozen at -80°C for further biochemical analysis. Some cement samples were fixed with 4% of neutral buffered formaldehyde for microscopy.

### Confocal laser scanning microscopy

Actin cytoskeleton and nuclei of the cells were stained by AlexaFluor 488® phalloidin and DAPI (both from Invitrogen), respectively, as described previously [18]. Imaging was performed using a Zeiss LSM confocal scanning laser microscope, located in the Core Facility Cellular Imaging (CFCI) of Technische Universität Dresden.

### TRAP staining

TRAP staining was performed for 10 min at 37°C by immersion of fixed samples in 0.3 mg/ml Fast Red Violet LB (Sigma) in 0.05 M sodium acetate (Sigma), 0.05 M acetic acid (Sigma), 0.03 M sodium tartrate (Roth), 0.1 mg/ml naphthol AS-MX phosphate disodium salt (Sigma) and 0.1% Triton X100 (Sigma). After washing with PBS, cell nuclei were stained for 10 min using Mayers Haemalaun solution (AppliChem) followed by rinsing in tap water. Stained samples were imaged using a Keyence BZ-9000 (Biorevo).

### Activity quantification of osteoclast-specific enzymes

Prior to determination of intracellular enzyme activities samples were thawed, cells were lysed using 1% Triton X-100 in PBS for 50 min on ice with a 10 min treatment in an ultrasonic bath. Cell lysates were collected in 0.5 ml tubes and subjected to the different assays.

#### TRAP activity

10 μl of cell lysate was mixed with 50 μl TRAP reaction buffer (2.5 mM naphthol-ASBI-phosphate*HCl (Sigma) in 100 mM sodium acetate and 50 mM disodium tartrate pH 6.1) and incubated at 37°C for 30 min. Finally, 150 μl of 0.1 M NaOH was added to stop the enzymatic reaction. The intensity of fluorescence was measured with a multifunction microplate reader at an excitation and emission wavelength of 405/520 nm. Calibrator solutions with different TRAP concentrations (0.6 to 12 U/L) taken from a commercially available ELISA kit (BoneTRAP, Medac) were used to correlate the fluorescence intensity with TRAP activity.

#### CA II activity

50 μl of cell lysate were mixed with CA II reaction buffer containing 12.5 mM TRIS pH 7.5, 75 mM NaCl and 2 mM 4-nitrophenylacetate (50 μl, Sigma). Absorbance was monitored at 400 nm for 5 min. Conversion to 4-nitrophenol was calculated from the slope of the absorbance plot using a calibration line from different dilutions of 1 mM 4-nitrophenol (Sigma).

#### CTSK activity

10 μl of cell lysate were diluted with lysis buffer to a final volume of 50 μl and 50 μl of substrate solution (0.1 mM Z-LR-AMC (Enzo Life Sciences) in 0.1 M sodium acetate buffer containing 4 mM EDTA and 4 mM DTT at pH 5.5) were added. After incubation for 30 min at 37°C the fluorescence intensity was measured at an excitation/emission wavelength of 365/440 nm. The amount of released aminomethylcoumarin (AMC) was quantified with a calibration line.

### Analysis of resorption pits on dentin discs

After performing cell lysis as described above, dentin samples were washed with deionized water and dried at 60°C for 12 hours. After drying, samples were mounted on stubs, sputter coated with gold and imaged using a Philips XL 30/ESEM with FEG (field emission gun), operated in SEM mode. Six independent SEM images per sample with 500x magnification were used for the calculation of resorption area applying the open source software Fiji [19]. The combined area of resorption pits and trails was calculated after manually marking with the oval selection tool.

### Gene expression of osteoclast markers

RNA was extracted from six cell- seeded cement samples of each sample group and for cells of two donors using the peqGOLD MicroSpin Total RNA Kit (Peqlab) according to the manufacturer’s instructions. During the RNA isolation procedure, cell lysates of three samples of each sample group were pooled to obtain a sufficient amount of RNA for further analysis. 100 ng of total RNA were transcribed into cDNA in a 20 μL reaction mixture containing 200 U of superscript II reverse transcriptase (Invitrogen), 0.5 mM dNTPs (Invitrogen), 12.5 ng/μL random hexamers (Eurofins MWG Operon, Germany) and 40 U of RNase inhibitor RNase OUT (Invitrogen). 1 μl cDNA in 20 μL reaction mixtures containing specific primer pairs was used for amplification in PCR analysis to detect transcripts of TRAP (forward TTC TAC CGC CTG CAC TTC AA; reverse AGC TGA TCT CCA CAT AGG CA), CA II (forward TAA CTT CGA TCC TCG TGG CCT C; reverse GCC AGT TGT CCA CCA TCA GTT C), CTSK (forward GAT ACT GGA CAC CCA CTG GGA, reverse CAT TCT CAG ACA CAC AAT CCA C) and vitronectin receptor (VNR) (forward AAG TTG GGA GAT TAG ACA GAG G, reverse CTT TCT TGT TCT TCT TGA GGT GG). GAPDH was used as housekeeping gene. Primer sequences were designed with “PCR Primer Design” (Eurofins). All synthesis steps were carried out in a thermocycler (Mastercycler®pro, Eppendorf). PCR products were visualised in 2% agarose gels (Ultra PureTMAgarose, Invitrogen). Intensity of the gel bands was analysed with Image J 1.44p (freeware, NIH, U.S.A.) and related to the intensity of GAPDH band.

### Release of inflammatory cytokines

PBMC were isolated from buffy coats of three different donors as described in the chapter “osteoclast formation”. Cement samples (3 mm diameter, 4 mm height, sterilized by γ-irradiation and pre-incubated in 2 ml of α-MEM containing 10% heat-inactivated FCS overnight) were seeded with 1*10^6^ PBMC per sample in 12 well plates, precoated with BSA to prevent protein binding to the dish. Four samples were prepared per donor and cement modification (reference, Cu10, Co10, Cr10 and Cr50). Furthermore, three samples per donor were seeded on TCPS without addition of cements (control) and three samples per donor were seeded on TCPS and stimulated with 1 μg/ml lipopolysaccharide (LPS) (positive control). All samples were cultivated in 2 ml α-MEM containing 10% heat-inactivated FCS. Supernatants were collected after 16 h of incubation and frozen at -20°C until further analysis. The concentrations of tumor necrosis factor alpha (TNF-α) and interleukin 1 beta (IL-1β) were determined using human TNF-α TMB ELISA development kit and human IL-1β TMB ELISA development kit (both from Peprotech) according to manufacturer`s instructions.

### Statistics

For the measurement of osteoclast-specific enzyme activities and gene expression analysis one-way analysis of variance (ANOVA) was used to evaluate statistical significance at a level of p<0.05. For the resorbed area two-way ANOVA with cement modification and donor as parameters was used to evaluate statistical significance at a level of p<0.05. Post-hoc analysis was performed in all cases to determine multiple comparisons using the Tukey method (Origin 9.1, OriginLab).

## Results

### Osteoclast formation on the cements

**Fig 1:**
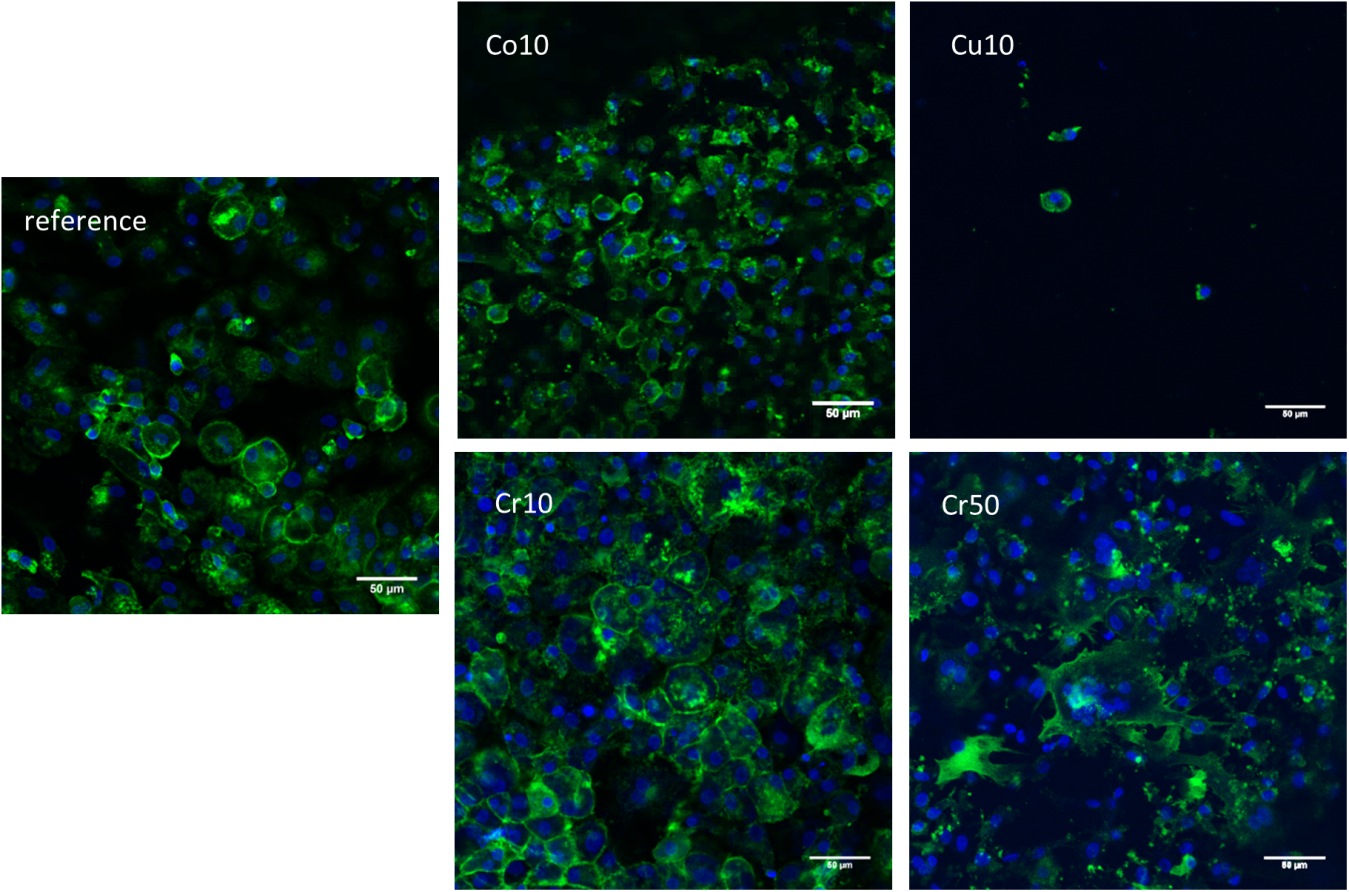
Osteoclast formation on the surface of the cements. CLSM images of osteoclasts differentiated from human PBMC for 16 days under stimulation with M-CSF and RANKL on brushite cement as reference, and on Cu10, Co10, Cr10 and Cr50 modifications. Nuclei are stained in blue, and actin cytoskeleton is stained in green. Scale bars represent 50 μm.

After 16 days of cultivation osteoclast-like cells with actin rings were detected on the reference cement and on the Co10, Cr10 and Cr50 modifications (Fig 1). Cells and actin rings were smaller on Co10 cement compared to reference and the Cr^3+^ doped cements. Only in some cases (PBMC of two donors) scattered cells were visible on the surface of Cu10 cements. When the cement discs were seeded with cells of three other donors, no cells were detectable on the surface of Cu10 cements.

### Osteoclast-specific enzyme activities

**Fig 2:**
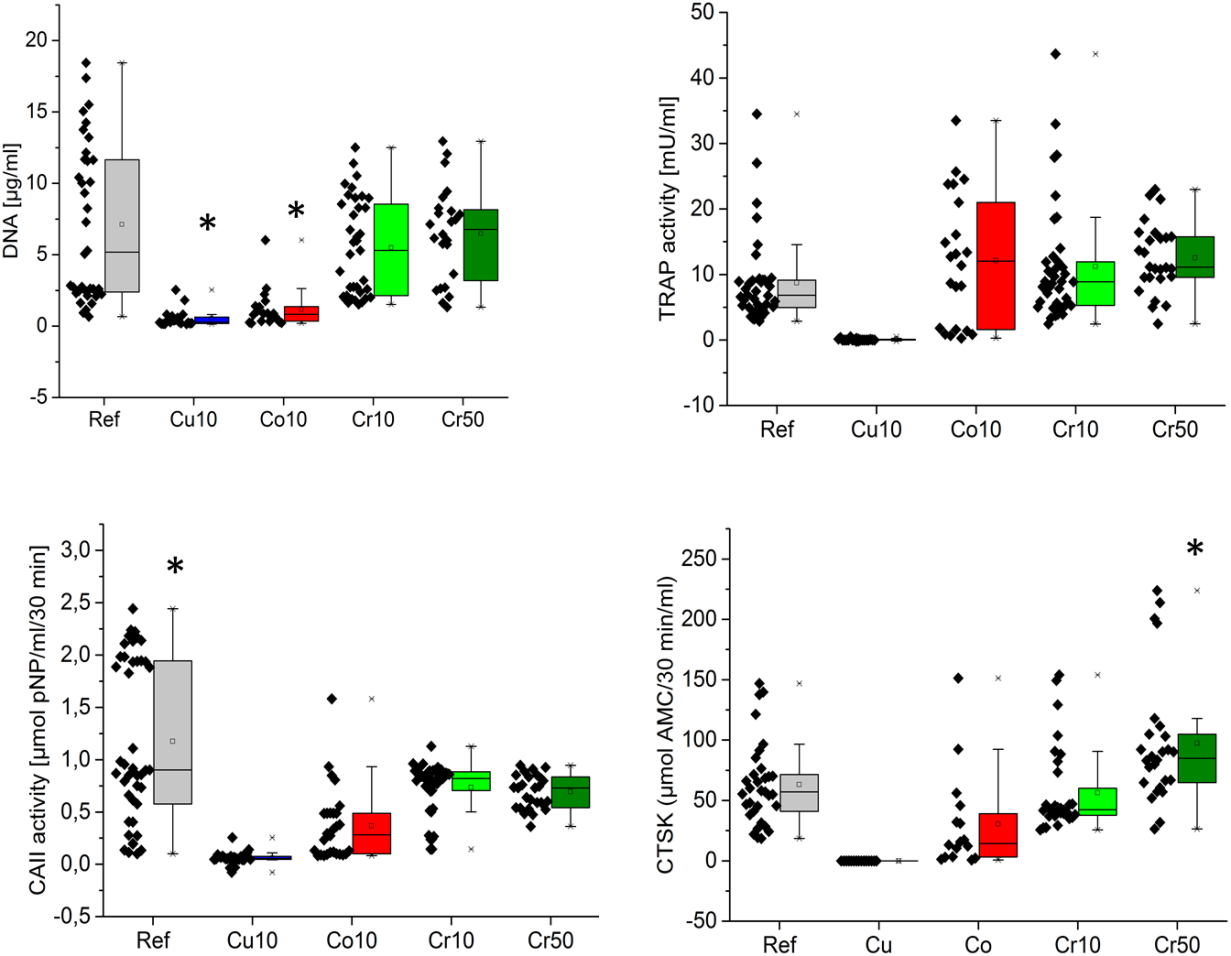
DNA content, TRAP, CAII and CTSK activities of osteoclasts after direct cultivation on CPC. Osteoclasts were differentiated for 16 days directly on the surface of the CPC (reference) and Cu10, Co10, Cr10 and Cr50 modifications. PBMC of 5–9 donors were included into the analysis. Each box shows the 25th to 75th percentile of the measured data. Squares (□) represent mean values, horizontal bars inside the box show the median value, while upper and lower bars indicate the upper and lower values within 1.5 times the inter-quartile range from the upper and lower quartile. Individual data points are shown on the left side of each box. * statistically significant differences (p<0.05) to all other sample types.

After 16 days of cultivation activity of TRAP, CAII and CTSK was detected on all cement modifications except Cu10 (Fig 2). There were no significant differences (p<0.05) between TRAP activities of the different cement modifications Co10, Cr10, Cr50 and reference cement. CA II activity on the reference cement was significantly (p<0.05) higher compared to all metal ion containing modifications. CTSK activity on Cr50 cement was significantly higher compared to all other modifications and to the reference. Generally enzyme activities showed high variations, especially between cells of different donors. DNA content on Cu10 and Co10 cements was significantly (p<0.05) lower compared to the other cement modifications and the reference.

### Ion release from cements during cultivation

The concentration of Cu^2+^, Co^2+^ and Cr^3+^ ions in the cell culture medium was determined after 1 day and during the last three days (d13-d16) of osteoclast cultivation by ICP-MS. For Co^2+^ and Cu^2+^ the initial release was up to tenfold higher compared to the release between d13 and d16 of cultivation. In contrast very low amounts of Cr^3+^ were released by the cements samples but the Cr^3+^ concentration was higher after 16 days of cultivation compared to the initial release after 1 day (Table 1).

**Table 1:** Release of metal ions from doped calcium phosphate cements. Cell culture supernatants from cement samples (6mm diameter, 1 mm height) during direct cultivation with human PBMC/osteoclasts were analysed by ICP-MS.

cement modification	metal ions released (mM) after 1 day	metal ions released between d13 and d16 (mM)
Cu10	0,305 ± 0,023	0,027 ± 0,003
Co10	0,247 ± 0,026	0,027 ± 0,003
Cr10	0,00102 ± 0,00002	0,00180 ± 0,00012
Cr50	0,00551 ± 0,00060	0,01057 ± 0,00062

**Fig 3:**
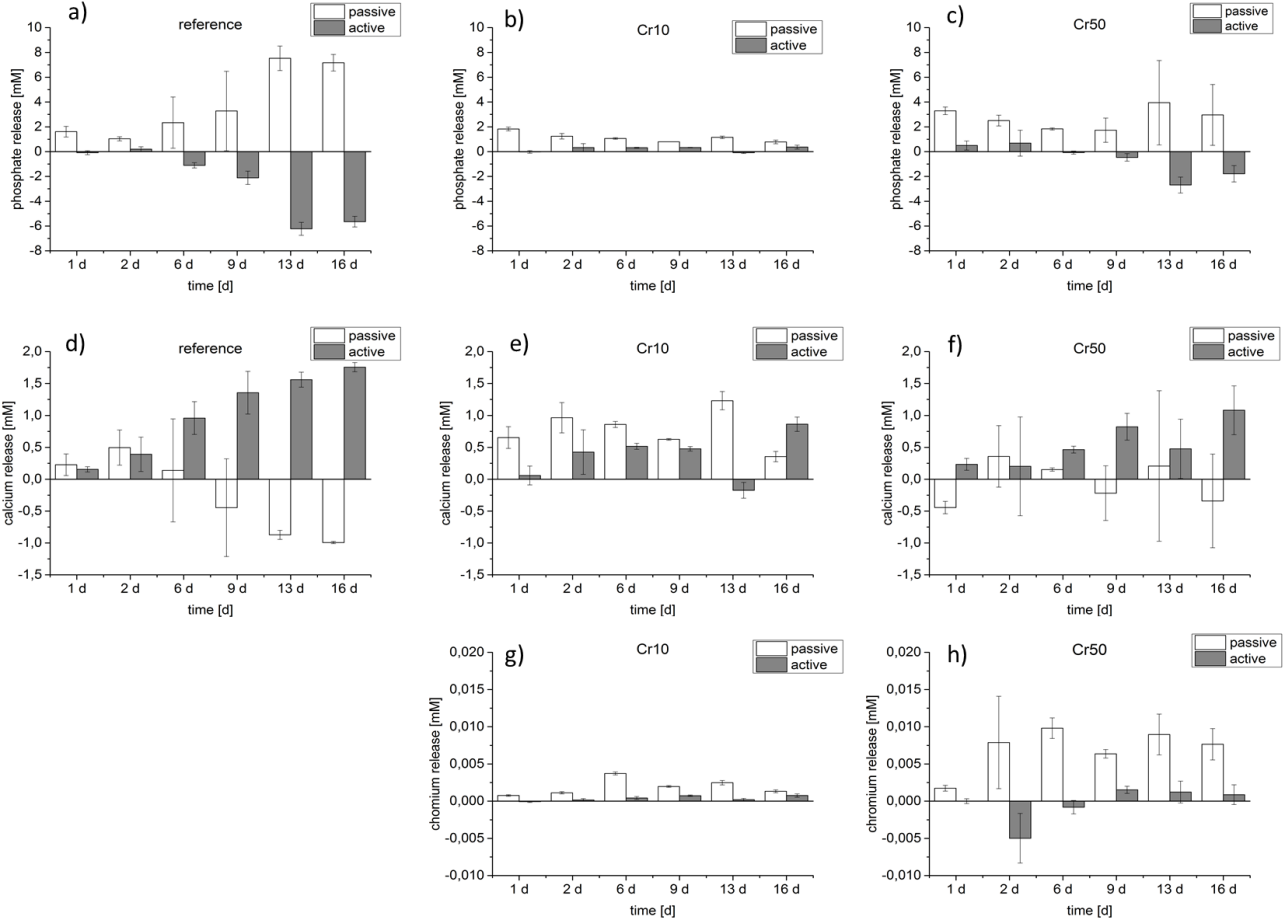
Release of PO43-, Ca^2+^ (d-f) and Cr^3+^ from Cr^3+^ doped CPC. ICP-MS was used to quantify the release of PO_4_^3-^(a-c) Ca^2+^ (d-f) and Cr^3+^ (g, h) into cell culture medium in the absence of cells (passive) and during cultivation of PBMC/osteoclasts (active). For passive release 6 samples of the respective cement modification were incubated in each 200 μl of cell culture medium. For active release PBMC of three different donors were seeded on 18 cement samples per modification in total (6 samples per donor). Medium of all samples was changed at days 1, 2, 6, 9, 13 and 16. Three medium samples per donor, modification and cultivation day were pooled to get enough volume for analysis. Cell culture medium concentration of Ca^2+^and PO_4_^3-^ was subtracted as blank.

In a second experiment the concentration of Ca^2+^, PO_4_^3-^ and Cr^3+^ released from Cr10 and Cr50 and reference cement samples in the absence of cells (passive ion release) and during cultivation of PBMC/osteoclasts (active release) was analysed by ICP-MS (Fig 3). Again an increase of Cr^3+^ concentration during cell cultivation was detected. Interestingly, the Cr^3+^ release was considerably higher in the absence of cells (Fig 3G and 3H).

The release of Ca^2+^ from the reference cement showed a steady increase over cultivation time in the presence of cells. In contrast Ca^2+^ concentration in the medium dropped below basal level from day 9 in the absence of cells (Fig 3D). Cr10 and Cr50 cements released lower Ca^2+^ amounts compared to the reference in the presence of cells. Interestingly, the Cr10 modification also released Ca^2+^ in the absence of cells (Fig 3E).

Phosphate release of the reference cement increased during incubation in the absence of cells, while it dropped below basal medium concentration in the presence of osteoclasts (Fig 3A). Cr10 and Cr50 cements released lower phosphate amounts compared to the reference in the absence of cells. In the presence of osteoclasts the phosphate content dropped, but not as drastically as during cultivation of osteoclasts on the reference cement (Fig 3B and 3C).

### Gene expression of osteoclast-specific markers

**Fig 4:**
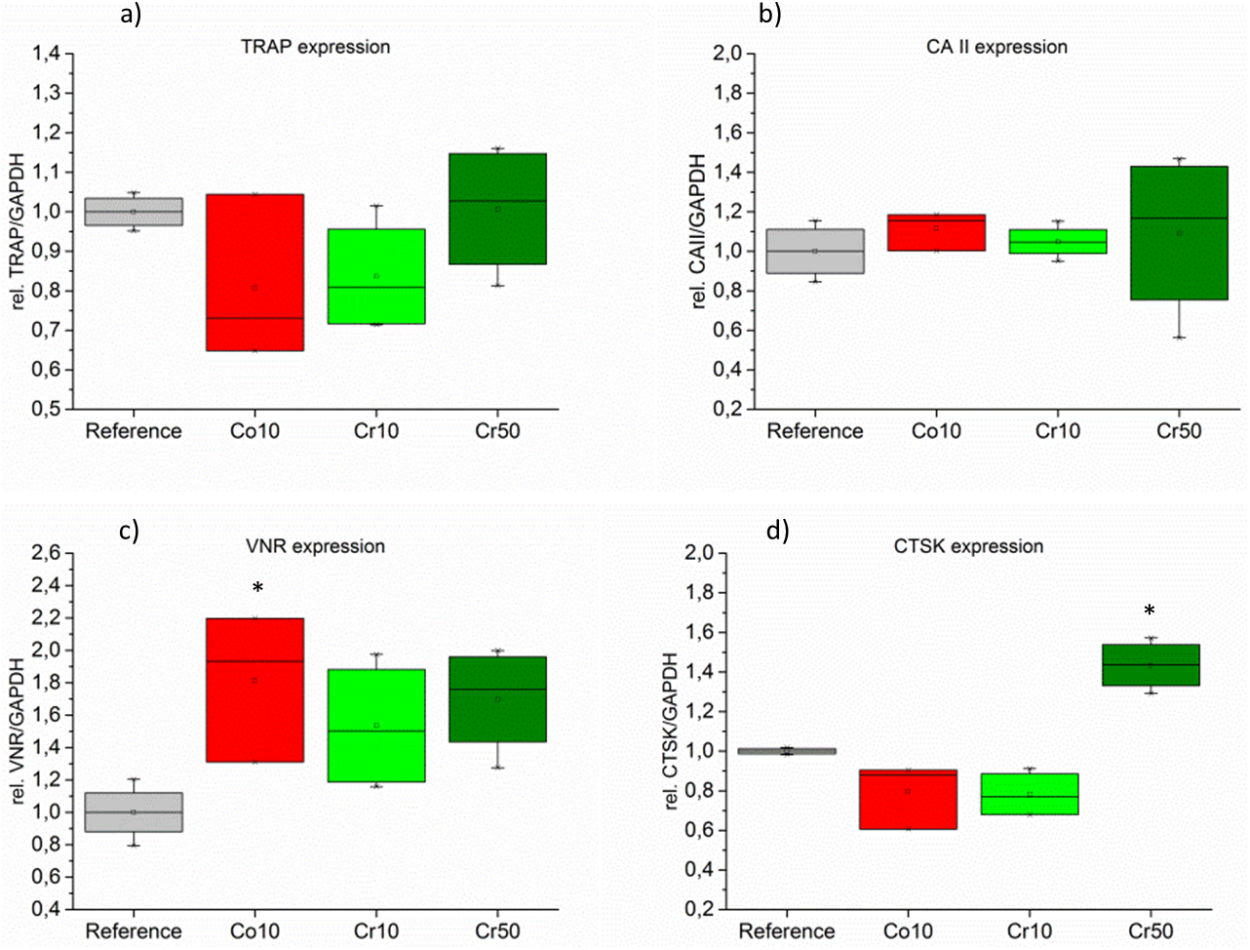
Gene expression analysis. Expression of osteoclast-specific genes was analysed after 16 days of osteoclast cultivation on CPC (reference) and Co10, Cr10 and Cr50 modifications A) TRAP, B) CAII, C) VNR and D) CTSK. Six cement samples of each modification were seeded with cells and lysates of three samples were pooled to obtain two data points per modification and donor. Cells of two donors were included into the analysis. PCR products were visualized in agarose gels; the intensities of the gel bands was analysed using the open source software Fiji and related to the intensity of the GAPDH bands. Furthermore, the band intensities of the reference cement were set to 1 for each individual donor. * statistically significant differences (p<0.05) to the reference.

Gene expression of typical osteoclast-related genes TRAP, CAII, VNR and CTSK was detected on the reference cement and the Co10, Cr10 and Cr50 modifications after 16 days of osteoclast cultivation. In the case of Cu10 cements, isolated RNA amounts were not sufficient for analysis. TRAP and CAII expression did not show significant differences between the cement modifications (Fig 4A and 4B). In contrast, the expression of VNR was higher in all modified cements compared to the reference however the differences were only statistically significant for the Co10 modification (Fig 4C). CTSK expression of osteoclasts cultured on the Cr50 modification was significantly increased compared to the reference and Co10 and Cr10 modifications (Fig 4D).

### Release of inflammatory cytokines

Concentrations of TNF-α in the LPS stimulated samples after 16 h of incubation were 622 ± 77 pg/ml; 742 ± 98 pg/ml and 1055 ± 251 pg/ml for the samples of three different PBMC donors. TNF-α concentration in control samples and also in the supernatants of the different cement samples was below 23,44 pg/ml which was the detection limit of the applied ELISA kit.

Concentrations of IL-1β in the LPS stimulated samples after 16 h of incubation were 125 ±36 pg/ml; 116 ± 13 pg/ml and 88 ± 35 pg/ml for the samples of three different PBMC donors. IL-1β concentration in control samples and also in the supernatants of the different cement samples was below 5,9 pg/ml which was the detection limit of the applied ELISA kit.

### Indirect cultivation with cement extracts for resorption analysis

**Fig 5:**
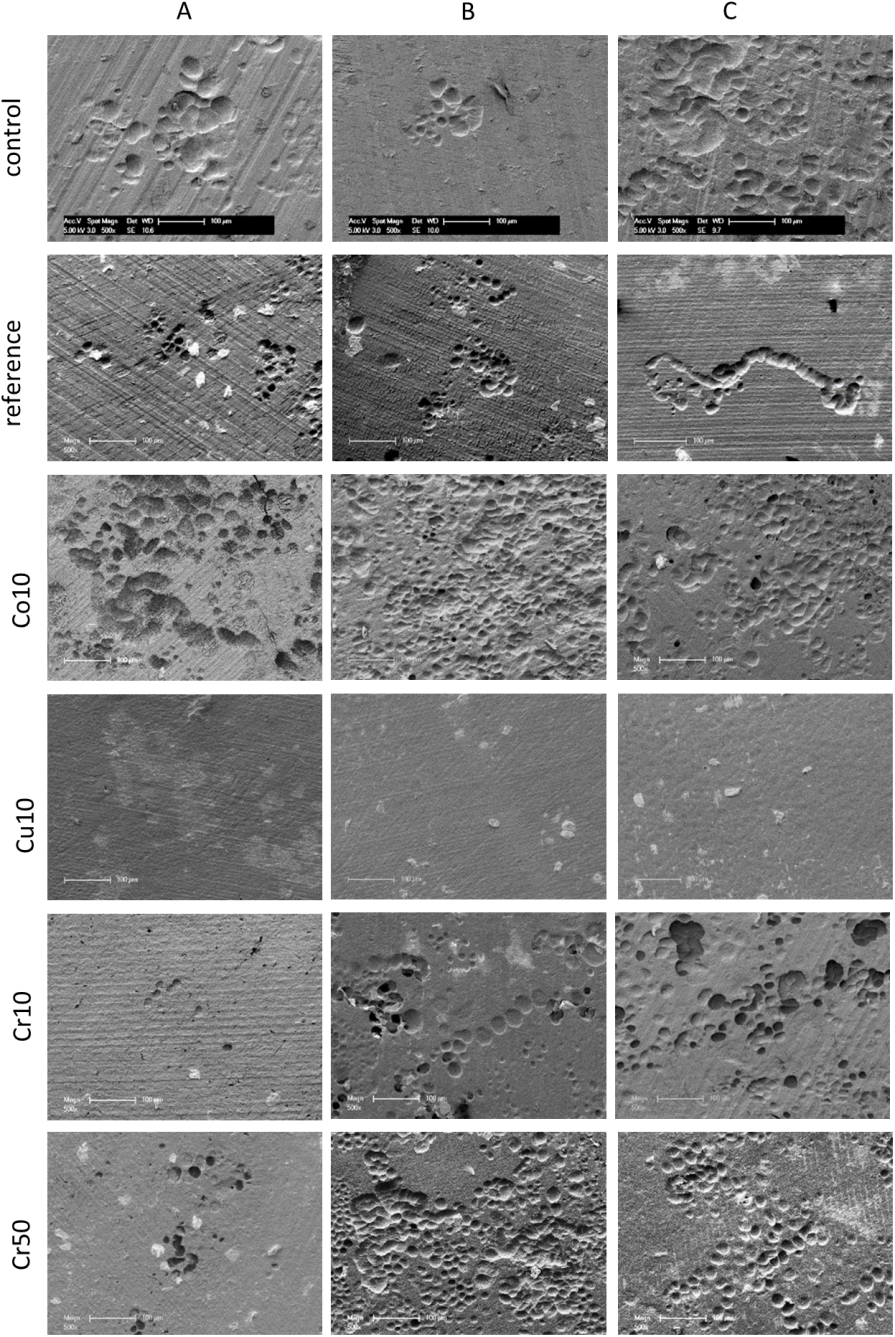
SEM analysis of resorption pits. Representative SEM images of resorption pits after 16 days cultivation of PBMC derived human osteoclasts on dentine slices in the presence of different cement extracts (reference = non-modified CPC) compared to cell culture medium (control). A, B, C represent cells of three different PBMC donors, scale bar represents 500 μm.

**Fig 6:**
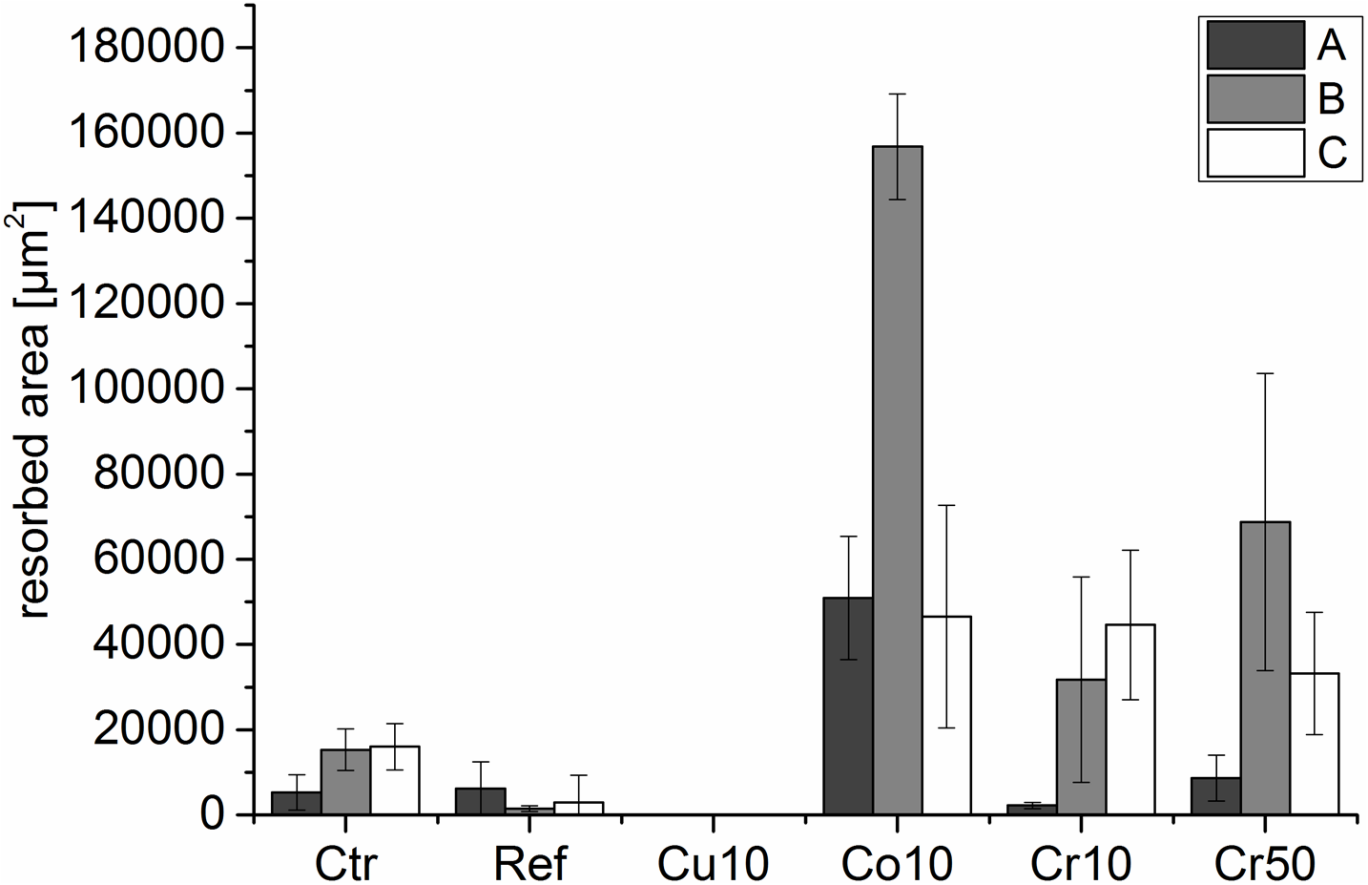
Average resorbed area on dentin slices. Osteoclasts were differentiated in the presence of different cement extracts (reference = non-modified CPC) compared to cell culture medium (control). Image J software was used to calculate resorbed area of six to eight SEM images with 500 x magnification, A, B, C represent cells of three different donors.

Since direct analysis of resorbed area was not possible on the examined cements because of their rough surface, PBMC were cultivated on dentine discs in the presence of extracts of the different cement modifications to analyse resorption activity. Extracts of Cu10 and Co10 cements were diluted with cell culture medium to avoid cytotoxic effects of Co^2+^ and Cu^2+^ (see materials and methods). Analysis of DNA content after 16 days of cultivation revealed donor-specific variations but no significant cell reduction compared to the control, indicating no cytotoxic effects of the applied cement extracts in the respective dilution (S1 Fig). Nevertheless, after 16 days of cultivation no resorption pits were detected on samples which were cultivated in the presence of Cu10 extracts. In contrast all other samples showed resorption pits (Fig 5). Resorbed area in the presence of unmodified cement extract was reduced in comparison to the control, however this effect was not statistically significant. Resorbed area in the presence of Co10, Cr10 and Cr50 extracts was significantly elevated compared to both reference and control (p<0.5) (Fig 6).

**Fig 7:**
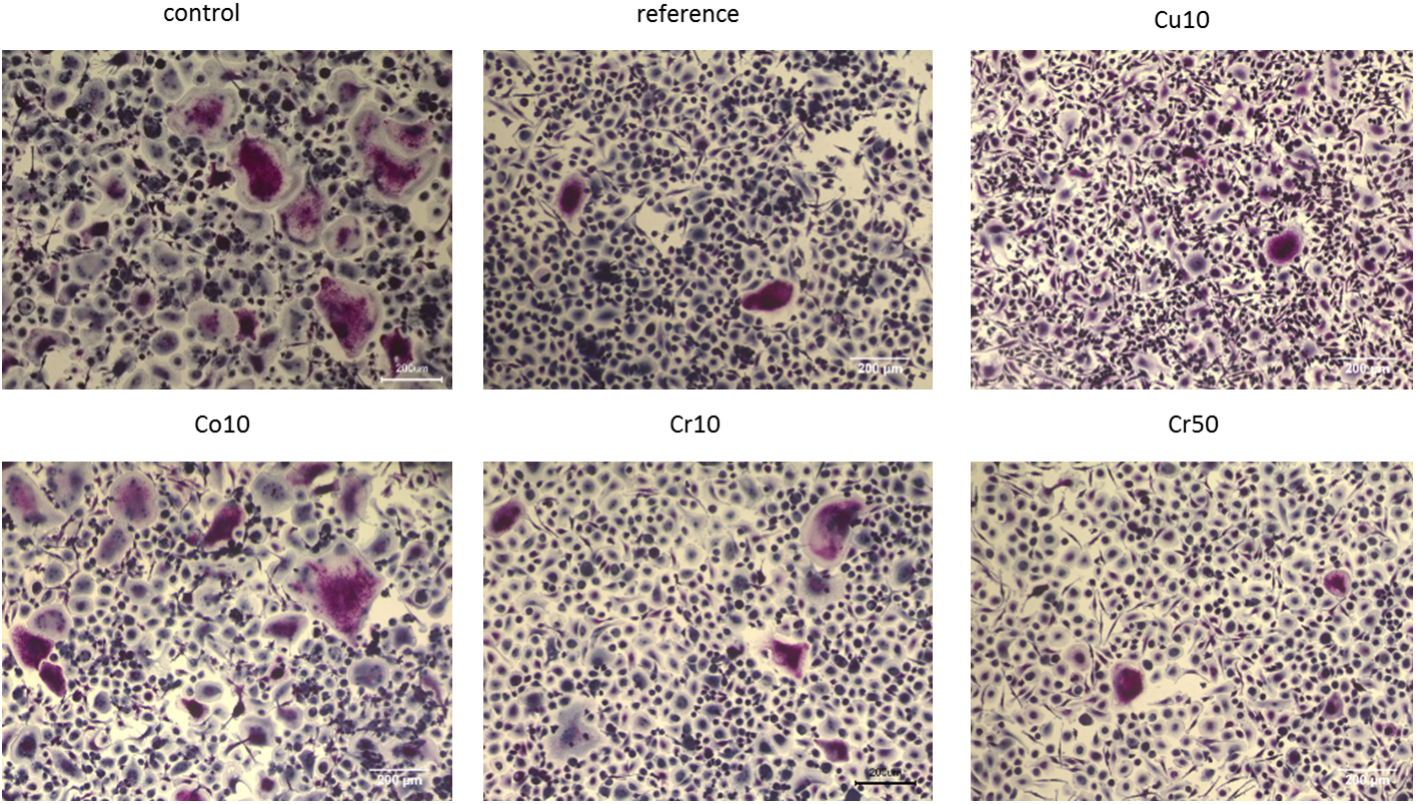
TRAP staining. Osteoclasts were differentiated in the presence of different cement extracts (reference = non-modified CPC) compared to cell culture medium (control) on TCPS for 16 days. TRAP activity stained in purple, nuclei are counterstained in blue. Scale bars represent 200 μm.

Additionally, PBMC were cultivated with cement extracts on TCPS under osteoclastic stimulation. After 16 days of cultivation, TRAP staining revealed the formation of TRAP positive multinucleated osteoclasts in all samples (Fig 7). Cells which were cultivated without cement extracts (control) and in the presence of diluted extracts from Co10 cements showed a higher number of osteoclasts and higher osteoclast size compared to all other samples. In contrast, osteoclast size and number was reduced in the presence of extracts from the reference cement, Cr10 and Cr50 cement and diluted Cu10 cements.

## Discussion

Bioactive metal ions are an inexpensive and stable alternative to growth factors for the modification of calcium phosphate based biomaterials. To evaluate the possible impact of Cu^2+^, Co^2+^ and Cr^3+^ ions on healing of bone defects, *in vitro* investigations on the impact of the modified materials on angiogenesis and osteogenesis, but also on osteoclast-mediated resorption are necessary. The aim of the present study was to analyse the impact of Cu^2+^, Co^2+^ and Cr^3+^ modification of calcium phosphate cements on osteoclast formation and activity. Osteoclast formation was observed on all cement modifications except Cu10 modification after 16 days of cultivation. Measurement of DNA content revealed very low cell number on Cu10 cements, so the failed osteoclast formation in these experiments was presumably caused by cytotoxic effects due to the burst release of Cu^2+^. The released concentration of Cu^2+^ after 1 day of incubation was 305 ± 23 μM, which is far above the cytotoxic threshold for PBMC/osteoclasts of approximately 30 μM (S2 Fig). Consequently, besides the low DNA content no activity of the osteoclast-specific enzymes TRAP, CAII and CTSK was detected for the Cu10 cement modification. To further evaluate the impact of Cu10 modification on resorption and to obtain an impression whether fabrication of Cu^2+^ doped cements with lower Cu^2+^ doses would be beneficial for application as bone graft material, extracts for the indirect resorption assay on dentin slices were diluted to a final Cu^2+^ concentration of 17.8 μM. Nevertheless, cultivation of PBMC under osteoclastic stimulation on dentin in the presence of the Cu^2+^ containing extracts revealed no resorption of dentin despite the survival of the cells which was demonstrated by DNA content of the samples which was in a similar range compared to all other examined samples (S1 Fig). Furthermore, TRAP staining on TCPS indicated the formation of TRAP positive multinucleated cells in the presence of diluted Cu10 extracts. We therefore postulate, that Cu^2+^, at least when released from a calcium phosphate cement rather inhibits the resorption activity of osteoclasts than their formation. Similar results were obtained by Yang and co-workers, who cultivated primary rabbit osteoclasts on calcium phosphate films with incorporated Cu^2+^ [15]. While the osteoclast number was not significantly affected by Cu^2+^ containing calcium phosphate films and no cytotoxic effects were revealed by determination of lactate dehydrogenase activity in the supernatants, the resorbed area of the calcium phosphate films significantly decreased with increasing Cu^2+^ concentration in the films. However, Cu^2+^ release into cell culture medium was not quantified in this study which makes it harder to compare these data. A historic investigation of Wilson and coworkers likewise revealed an inhibiting effect of Cu^2+^ on bone resorption [20]. It was shown, that 10 μM Cu^2+^ decreased resorption to 33% of control samples measured by release of ^45^Ca. Cu^2+^ doped brushite cement was included into our study since Cu^2+^ doping of calcium phosphate ceramics was shown to accelerate and guide angiogenesis [6]. The inhibited resorption of Cu^2+^ containing calcium phosphate cements might be beneficial for patients which show a impaired balance between osteogenesis and resorption, e.g. during osteoporosis. Since the presence of viable osteoclasts is important for cross-talk of bone cells during remodeling, Cu^2+^ doped cements should contain in future considerably less Cu^2+^ than the Cu10 cement used in the present study.

Co10 cements showed a burst release of Co^2+^ with 247 ± 26 μM Co^2+^ present in the supernatant after 1 day of incubation in cell culture medium. We did not specifically analyse the cytotoxic threshold of Co^2+^ for PBMC/osteoclasts, but previous investigations have shown a slight cyctotoxic effect of 100 μM Co^2+^ and a strong cytotoxic effect of 500 μM Co^2+^ (S3 Fig). The observed burst release of Co^2+^ was also previously detected by examining brushite cements of different dimensions [7] and also described during release of Co^2+^ from calcium phosphate coatings [21]. The relatively high initial Co^2+^ concentration might be the reason for the drastically reduced DNA content of cell lysates after 16 days of cultivation of PBMC/osteoclasts on Co10 cements compared to the reference cement. Nevertheless osteoclasts, visible as multinucleated cells with actin rings were detected also on the surface of Co10 cements. Furthermore TRAP activity of the cell lysates after cultivation of osteoclasts on Co10 was not significantly different to TRAP activity in the presence of the reference cement and the Cr^3+^ modified cements. TRAP activity has been established as a marker for osteoclast number at least on TCPS and bone [22,23]. In a previous study we have comprehensively investigated the activity of osteoclast specific enzymes TRAP, carbonic anhydrase II (CA II) and cathepsin K for human osteoclasts on different materials [17]. A correlation between intracellular TRAP and CAII activity and resorbed area was demonstrated for hydroxyapatite forming calcium phosphate bone cements. Intracellular TRAP activity on Co10 cement therefore suggests an active resorption of the material despite low cell number. Gene expression of vitronectin receptor, a late stage osteoclast differentiation marker was significantly increased for osteoclasts on Co10 cements further evidencing a promoting effect of Co10 cement on osteoclastogenesis. Indirect resorption assays on dentine slices in the presence of Co10 cement extracts resulted in the highest resorbed area compared to all other cement extracts. These results suggest a clear resorption promoting effect of Co^2+^ especially in concentrations around 20 μM and in the presence of calcium phosphate bone cement. Similar results were obtained after cultivation of bone marrow derived mouse osteoclasts on calcium phosphate coatings doped with Co^2+^ [21]. TRAP activity and total resorbed area were elevated in the presence of Co^2+^ while osteoclast number was only increased at very low Co^2+^ concentrations (0.1 μM when added to the medium and 1 μM incorporated into the calcium phosphate layer). Two more studies analysed the effect of Co^2+^ without additional biomaterials on human PBMC derived osteoclasts. Andrews and co-workers [14] report only a mild stimulatory effect of Co^2+^ on developing osteoclast number and resorption activity at very low concentrations (around 0.01 μM). Higher Co^2+^ concentrations (10–200 μM) decreased both osteoclast number and resorbed area for developing and mature osteoclasts. In contrast Mabilleau et al [24] report a significant increase of both number and size of multinucleated TRAP positive osteoclasts after cultivation of PBMC derived human osteoclasts in the presence of 100 μM Co^2+^. Due to the significantly increased osteoclast number at 100 μM Co^2+^, the resorbed area per osteoclast was strongly decreased. Evidently the effect of Co^2+^ on osteoclastogenesis and resorption depends on several factors like donor variability of the primary cells, presence (or absence) of other ions like Ca^2+^ and PO_4_^3-^ in combination with calcium phosphate based materials and variations of concentration during Co^2+^ release. Beside the partly contradicting data from the literature, the complex effect of Co^2+^ also becomes obvious in the present study: Co10 cements seem to show no enhanced resorption compared to non-modified calcium phosphate cements (as deduced from TRAP and CAII activity), but osteoclast cultures on dentine with diluted extracts from Co10 cements showed a considerably increased resorbed area, which was confirmed independently by cells of three different donors. Nevertheless the modification of brushite cements with Co^2+^ for better remodeling of bone defects seems to be not promising because of its cytotoxic effects towards osteoblast progenitor cells as shown in our previous study [7]. Furthermore, Co^2+^ incorporated β-TCP has been shown to switch the macrophage phenotype to M1 which is related to the release of pro-inflammatory cytokines [25]. However, in the present study PBMC cultured in the presence of Co^2+^ doped cements did not show elevated levels of the pro-inflammatory cytokines TNF-α and IL-1β.

The incorporation of Cr^3+^ into brushite forming cements resulted in the formation of barely soluble chrome phosphates which was accompanied by release of very low concentrations of Cr^3+^ compared to Co^2+^ and Cu^2+^ [7]. In the present study the low and steady release of Cr^3+^ was confirmed. Interestingly and unexpectedly the Cr^3+^ release in the presence of PBMC/osteoclasts was lower compared to the passive release in the absence of cells. Possibly, Cr^3+^ was endocytosed by the cells as shown previously for PBMC derived human osteoclasts [26]. Osteoclast formation on the Cr^3+^ doped cements was similar to the reference and to the Co^2+^ doped modification. Also the activity of osteoclast-specific enzymes was comparable to the reference and the Co^2+^ doped modification. However, the activity of CTSK was significantly increased for osteoclasts on Cr50 compared to the reference and both Cr10 and Co10. Coincidently, the gene expression of CTSK was significantly higher on Cr50 compared to all other modifications. Taking a look to the results of the indirect resorption tests it becomes obvious that, at least for two of the three examined donors, the resorbed area was considerably increased with Cr50 extracts compared to the reference and control. Due to the very low Cr^3+^ concentration compared to Co^2+^ and Cu^2+^ (highest values for Cr^3+^ release in the presence of osteoclasts was ~0.7 μM for Cr10 and ~1 μM for Cr50 cements) no cytotoxic effects are expected. Even concentrations of 1mM Cr^3+^ did not provoke any cytotoxic signs in PBMC/osteoclasts (S4 Fig). Cr^3+^ concentration in the synovial fluid of patients with metal on metal prosthetic implants was found to be up to 25 μM [27]. At those and higher Cr^3+^ concentrations decreased osteoclast numbers (100 μM Cr^3+^) [24] and an inhibited osteoclastic resorption (200 μM Cr^3+^) [14] were detected. These Cr^3+^ concentrations are at least 2–3 magnitudes higher than the released Cr^3+^ concentration from the calcium phosphate cements in our study. Very low Cr^3+^ concentrations are reported to positively influence osteoclastogenesis and resorption. Andrews et al. reported an increased osteoclast number as well as an increased resorption after treatment of PBMC derived osteoclasts with 0,1 μM Cr^3+^ [14]. The possibly increased resorption of the Cr^3+^ doped cements could be caused not only by the released Cr^3+^ ions but also by the changed morphology of the cement matrix and the altered cement composition showing a significant amount of monetite as additional calcium phosphate phase in the case of Cr50 cements [7]. It has been demonstrated before that both brushite and monetite are actively resorbed by osteoclasts [28,29]. Furthermore, incorporation of Cr^3+^ into the calcium phosphate cement caused changes in the release/uptake of Ca^2+^ and PO_4_^3-^. The unmodified cement decreased the Ca^2+^ content of the incubation medium probably due to re-precipitation of hydroxyapatite when incubated without cells. Incubation in the presence of osteoclasts lead as anticipated to an ascending release of additional Ca^2+^ into the cell culture medium. Ca^2+^ release is commonly accepted as a marker for osteoclastic resorption of bone [30] and calcium phosphate bone cements [28,29,31]. Doping of brushite cements with Cr^3+^ prevented the uptake of Ca^2+^ and the release of PO_4_^3-^ suggesting a changed dissolution behavior of the cement modification. Ca^2+^ release from Cr^3+^ doped cements in the presence of osteoclasts was lower compared to the release from the reference cement indicating a lower resorption of the Cr^3+^ doped cement on the first glance. Nevertheless, this is in contrast to the results of osteoclast enzyme activities and gene expression of osteoclast markers on Cr^3+^ modified cements and to the indirect experiment with Cr10 and Cr50 cement extracts, suggesting higher resorption compared to the reference cement. We propose that Ca^2+^ release is no reliable resorption marker in the case of Cr^3+^ doped cements, since the dissolution/precipitation behavior of the brushite cement is changed by the Cr^3+^ modification.

The present study together with our previous study on osteoprogenitor cells revealed high cytocompatibility of Cr^3+^ doped brushite cements. A further crucial aspect for the evaluation of innovative biomaterials is the immunogenicity of the material, since inflammatory reactions may result in implant failure. Therefore, the release of the pro-inflammatory cytokines TNF-α and IL-1β was analysed for PBMC cultivated in the presence of metal ion doped cements and the reference cement. While LPS stimulated PBMC showed considerably increased cytokine levels, which were similar to already published data [32] the concentration of both TNF-α and IL-1β in the presence of metal ion doped and undoped brushite cements was below the detection limit of the assay. It has been shown earlier, that human monocytes (THP-1 cell line) were not stimulated to release TNF-α in the presence of different calcium phosphates (including brushite, HA and β-TCP) [33]. The finding that both pure brushite cement and Cr^3+^ doped brushite cement did not provoke enhanced release of pro-inflammatory cytokines from PBMC *in vitro* suggests high potential of these materials for bone regeneration *in vivo*.

## Conclusions

Cu^2+^ and Co^2+^ doping of brushite cements with 10 mmol/ g β-TCP may cause cytotoxic reactions towards osteoclasts and osteoclast progenitors during initial burst release of Cu^2+^ and Co^2+^ respectively. Cements with lower doses Cu^2+^ might be beneficial for application in bone regeneration, since Cu^2+^ at 18 μM completely inhibited resorption (but not osteoclast formation) which might be beneficial for patients with osteoporosis and therefore an imbalance between bone formation and resorption. Best results were obtained for brushite cements doped with different concentrations of Cr^3+^. Our study revealed an increased osteoclastic resorption of these cements compared to the non-modified brushite cement and a very low release of metal ions which diminishes the risk of cytotoxic reactions. Furthermore, Cr^3+^ doped brushite cements have been shown to increase viability of osteoprogenitor cells in comparison to non-modified brushite cement [7]. Therefore Cr^3+^ doped brushite cements are suggested as promising new material for application in bone regeneration.

## Supporting information

S1 Fig DNA content of PBMC/osteoclasts after 16 days of indirect cultivation with cement extracts. Osteoclasts were differentiated in the presence of different cement extracts (reference = non-modified CPC) compared to cell culture medium (control) on TCPS for 16 days. DNA was quantified from cell lysates (n = 5 per sample group and donor). A, B, C represent cells of three different donors.
(TIF)

S2 Fig Cytotoxic threshold for Cu^2+^. PBMC of two different donors were cultivated under standard conditions with 25 ng/ml MCSF and 50 ng/ml RANKL under addition of different concentrations of Cu^2+^ as Cu(NO_3_)_2_·3 H_2_O. After 16 days of cultivation, cells were fixed and stained for TRAP activity. Scale bars represent 100 μm.
(TIF)

S3 Fig Cytotoxic threshold for Co^2+^. PBMC of two different donors were cultivated under standard conditions with 25 ng/ml MCSF and 50 ng/ml RANKL under addition of different concentrations of Co^2+^ as Co(NO_3_)_2_·6 H_2_O. After 9 days of cultivation, cells were fixed and stained for TRAP activity. Scale bars represent 100 μm.
(TIF)

S4 Fig Cytotoxic threshold for Cr^3+^. PBMC of two different donors were cultivated under standard conditions with 25 ng/ml MCSF and 50 ng/ml RANKL under addition of different concentrations of Cr^3+^ as Cr(NO_3_)_3_·9 H_2_O. After 9 days of cultivation, cells were fixed and stained for TRAP activity. Scale bars represent 100 μm.
(TIF)
