# Supplementary figures and images for: Osteoclastic differentiation and resorption is modulated by bioactive metal ions Co2+, Cu2+ and Cr3+ incorporated into calcium phosphate bone cements
Source: PLoS One. 2017 Aug 1;12(8):e0182109. doi: 10.1371/journal.pone.0182109 (PMC5538673; doi:10.1371/journal.pone.0182109)

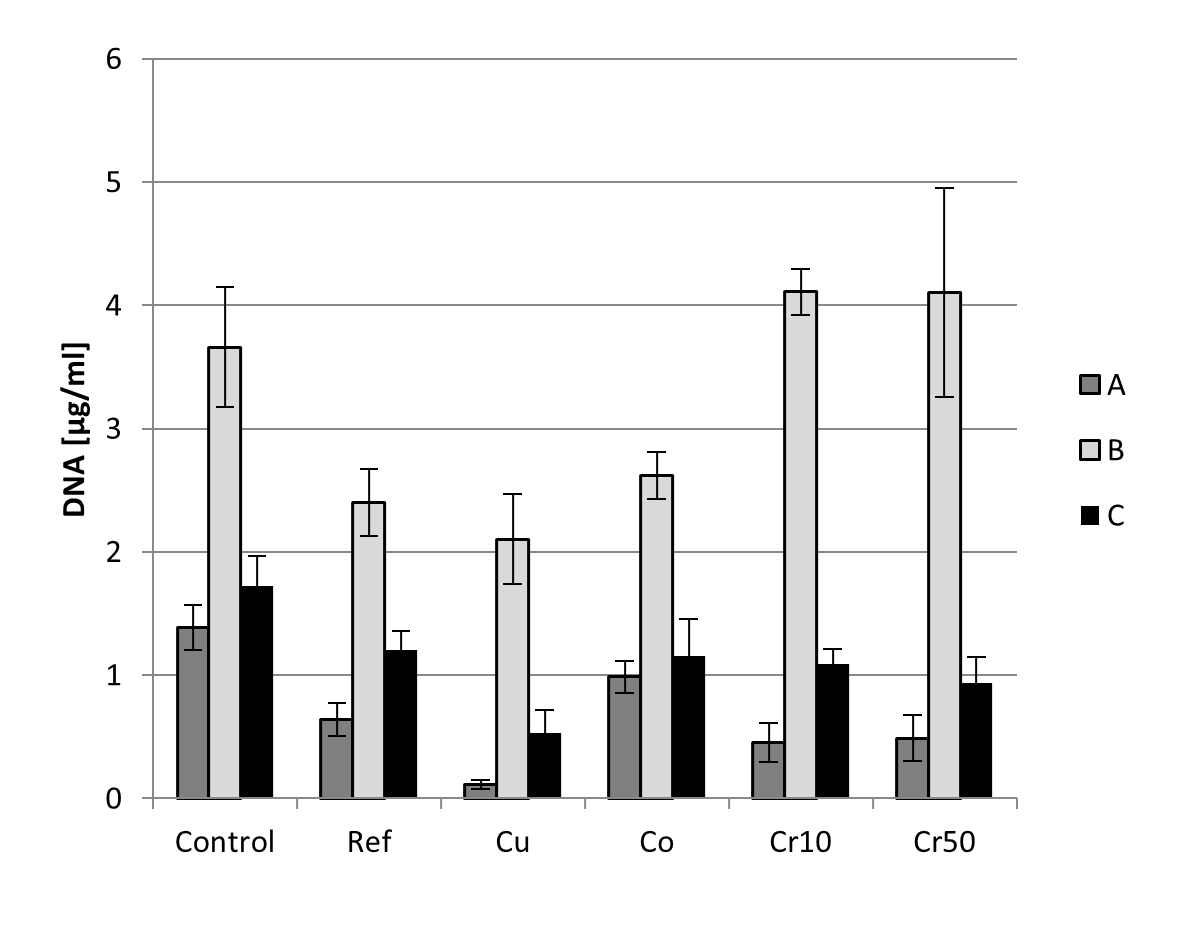

Supplement: S1 Fig — Osteoclasts were differentiated in the presence of different cement extracts (reference = non-modified CPC) compared to cell culture medium (control) on TCPS for 16 days. DNA was quantified from cell lysates (n = 5 per sample group and donor). A, B, C represent cells of three different donors. (TIF) [file pone.0182109.s001.tif]

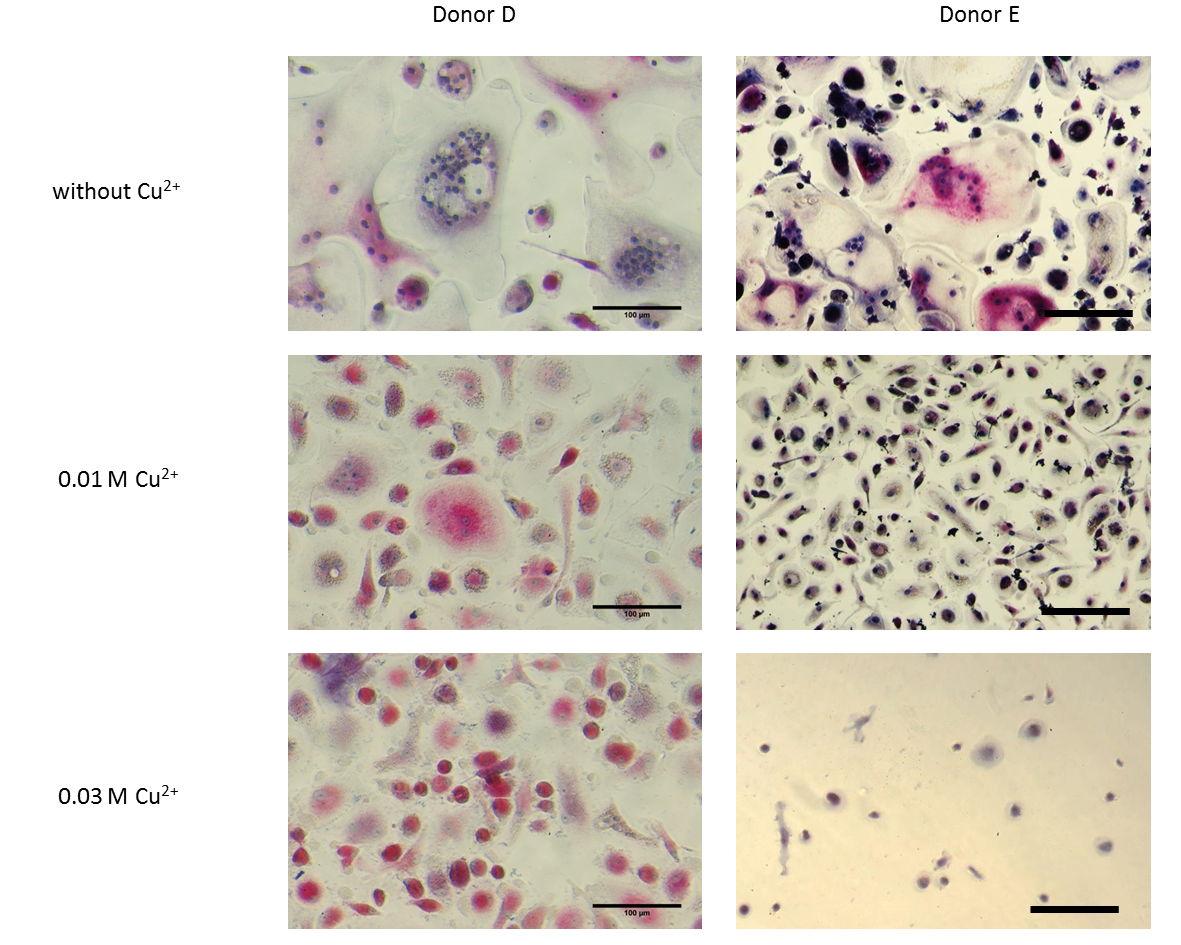

Supplement: S2 Fig — PBMC of two different donors were cultivated under standard conditions with 25 ng/ml MCSF and 50 ng/ml RANKL under addition of different concentrations of Cu2+ as Cu(NO3)2·3 H2O. After 16 days of cultivation, cells were fixed and stained for TRAP activity. Scale bars represent 100 μm. (TIF) [file pone.0182109.s002.tif]

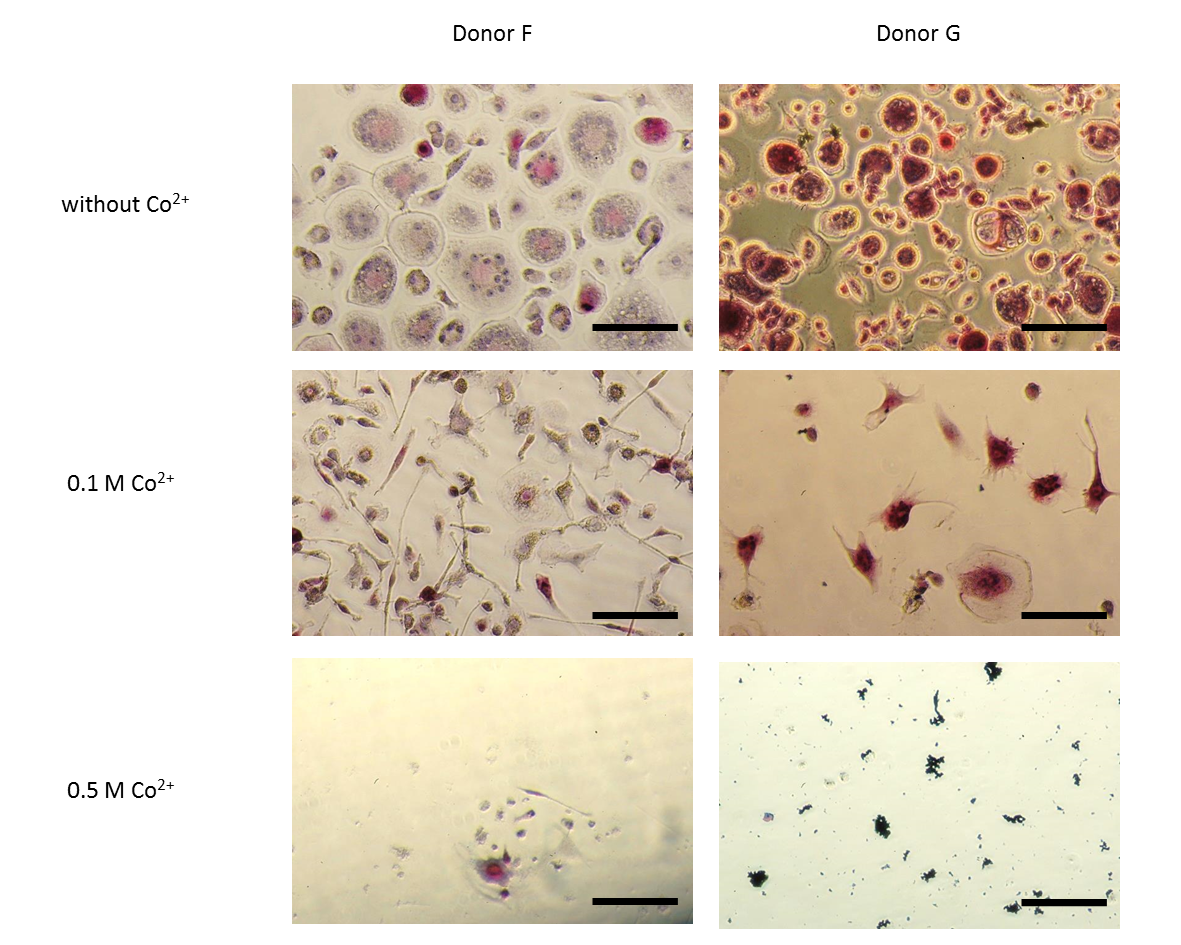

Supplement: S3 Fig — PBMC of two different donors were cultivated under standard conditions with 25 ng/ml MCSF and 50 ng/ml RANKL under addition of different concentrations of Co2+ as Co(NO3)2·6 H2O. After 9 days of cultivation, cells were fixed and stained for TRAP activity. Scale bars represent 100 μm. (TIF) [file pone.0182109.s003.tif]

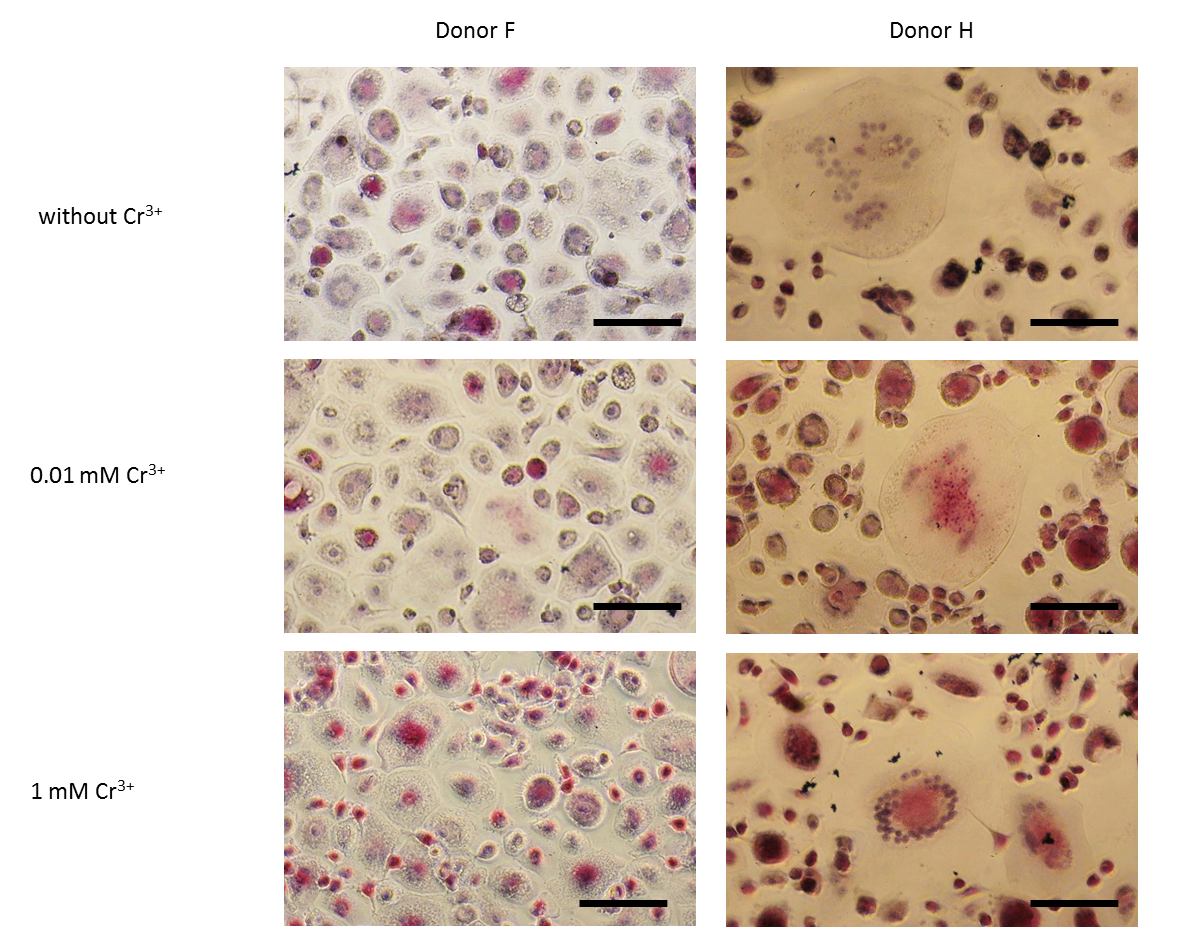

Supplement: S4 Fig — PBMC of two different donors were cultivated under standard conditions with 25 ng/ml MCSF and 50 ng/ml RANKL under addition of different concentrations of Cr3+ as Cr(NO3)3·9 H2O. After 9 days of cultivation, cells were fixed and stained for TRAP activity. Scale bars represent 100 μm. (TIF) [file pone.0182109.s004.tif]
